# Reliability of distally based sural flap in elderly patients: comparison between elderly and young patients in a single center

**DOI:** 10.1186/s12893-021-01175-6

**Published:** 2021-03-28

**Authors:** Ping Peng, Zhonggen Dong, Jianwei Wei, Lihong Liu, Zhaobiao Luo, Shu Cao

**Affiliations:** Department of Orthopedics, The Second Xiangya Hospital, Central South University, No. 139 Renmin Road, Changsha, 410011 Hunan China

**Keywords:** Elderly patients, Reconstruction, Soft-tissue defect, Sural flap, Surgical flap

## Abstract

**Background:**

Reconstructions the soft-tissue defects of the distal lower extremities in the elderly patients (≥ 60 years old) are full of challenges because of many comorbidities. The purpose of this study was to report the clinical application of the distally based sural flap in the elderly patients, and to verify the reliability of this flap in the elderly patients.

**Methods:**

Between March of 2005 and December of 2019, 53 patients aged over 60-year-old and 55 patients aged 18 to 30-year-old who underwent the procedure have been included in this study. The reconstruction outcomes, medical-related complications, flap viability-related complications and potential risk factors are compared between the group A (≥ 60 years old) and group B (ranging from 18 to 30 years old).

**Results:**

The partial necrosis rate in group A (9.43%) is higher than group B (9.09%), but the difference is not significant (P > 0.05). The constitute ratio of the defects that were successfully covered using the sural flap alone or combining with simple salvage method (i.e., skin grafting) is 96.22% and 98.18% in group A and B, respectively (P > 0.05). The differences of the risk flaps factors that affected the survival of distally based sural flap were not significant between group A and B (P > 0.05).

**Conclusions:**

The distally based sural flap can be effectively used to repair the soft-tissue defect of the lower extremity in the elderly patients. It is safe and reliable to harvest and transfer the flap in one stage, and the delay surgery is not necessary.

## Background

The soft-tissue defects of the distal lower extremities are common in the elderly patients, and the reconstructions of these defects are full of challenges because of many comorbidities. Many flaps, such as free flaps [1], perforator flaps [2], myocutaneous flaps [3], and regional pedicled flaps [4] have been used to repair the defects in such areas, and each has its own advantages, disadvantages and indications. Free flaps are considered by many surgeons to be the gold standard for reconstruction of the soft tissue defects [1, 5–7]. However, due to the complexity of the operation and the need for longer operation and anesthesia durations, the elderly patients are subjected to more postoperative complications [6–9]. The pedicled flap, with shorter duration in the operation scene are preferred to be employed to repair the distal lower extremity defect in the elderly patients.

The distally based sural flap is one of the mainstay flaps to reconstruct the soft-tissue defect over these areas [10–17]. Because of its rich blood supply, relatively simple procedure and reliable survival, this flap has been world widely popularized in the reconstructive surgery. Most of the studies on the distally based sural flap are targeted at the young and healthy patients [11–13]. The application of the flap with a relatively large sample in the elderly patients has not been reported in the literature. Many studies have shown that the advanced age (> 60 years old) is an independent risk factor affected the partial necrosis of the distally based sural flap, and the survival of the flap is also threatened by long-term adverse lifestyle (i.e., abuse of cigarette and alcohol) and medical comorbidities (i.e., hypertension, diabetes, atherosclerosis, and so on) in the elderly patients [14–16]. The questions of both whether the distally based sural flap can be applied in the elderly patients safely and reliably, and how to improve the flap viability in the elderly patients have not been elucidated in the publications. The purpose of this study was to report the clinical application of the distally based sural flap in the elderly patients, and to verify the reliability of this flap in the elderly patients by comparing with the young patients aged 18–30 years who have the best physical condition without any comorbidity during the same period.

## Patients and methods

There had been 435 distally based sural flaps with multifarious styles harvested in our department for reconstructions of the defects in the distal lower leg, ankle and foot from 2001 to 2020. All procedures were performed or supervised by the senior author. Between March of 2005 and December of 2019, 53 patients aged over 60-year-old and 55 patients aged 18 to 30-year-old who underwent the procedure had been included in this study. Patients in the two age groups were excluded if they received perforator-pedicled sural flaps. Data were retrogradely reviewed with regard to the patients demographics (i.e., sex and age), pathogenesis, location and size of the defect, medical comorbidities (including peripheral vascular disease, hypertension and diabetes mellitus), long-term adverse lifestyle (including nicotine intake and alcohol abuse), flap parameters (including the pivot point site, length and width of the fascial pedicle, length and width of the skin island, total length of the flap, length–width ratio [LWR] and top-edge of the flap) and outcomes of the reconstruction.

### Grouping and evaluation parameters

The flaps are divided into group A (≥ 60 years old) and group B (ranging from 18 to 30 years old) according to the age of the patients. The reconstruction outcomes, medical-related complications (including perioperative period death, pulmonary infection, deep vein thrombosis, pulmonary embolism, heart attack), flap viability-related complications (partial flap necrosis) and potential risk factors are compared between both groups.

The completely survival flap refers to the flap which survives completely or develops marginal necrosis no more than 1 cm in length, whereas the partial necrosis flap is defined as the flap which is necrotic in the border no less than 1 cm in length.

### Surgical procedure

The surgical procedure has been described in detail in the previous studies [10–17] and only some key features are highlighted here.

It is crucial for the elderly patients that evaluating the general body state and vascular condition of the extremities. Preoperative color ultrasonic Doppler are routinely utilized to not only locate a robust peroneal artery perforator closest to the defect, but also confirm the condition of the artery and vein in the lower extremities.

The flap is raised underneath the deep fascial layer with the Anterograde–Retrograde method [17] to keep a sizable peroneal artery perforator at the base of the fascial pedicle. The pivot point can be adjusted readily to the location of the perforator that closest to the defect to decrease the length of the fascial pedicle and the total length of the flap.

After the flap transposition, the affected limb was immobilized and elevated within 7 days, but the patients were instructed to perform muscle isometric contractions of the limb. When the flap was used to reconstruct the defect locating at the distal lower leg, the patients were instructed to exercise the affected limb at 14 days to 21 days postoperatively. However, when the defect was located at the foot and ankle, the walking exercise should be postponed to one month postoperatively.

### Statistical analysis

The SPSS 26.0 statistical software package is used to analyze the data. The continuous variables data were expressed as mean ± standard deviation ($\stackrel{-}{x}$ ± s), using t test to analyze the data. The categorical variables data was expressed by rate (constituent ratio), and Chi-square test or Fisher exact test was used to analyze the data. Results with P value < 0.05 indicate that the difference is statistically significant.

## Results

**Fig. 1:**
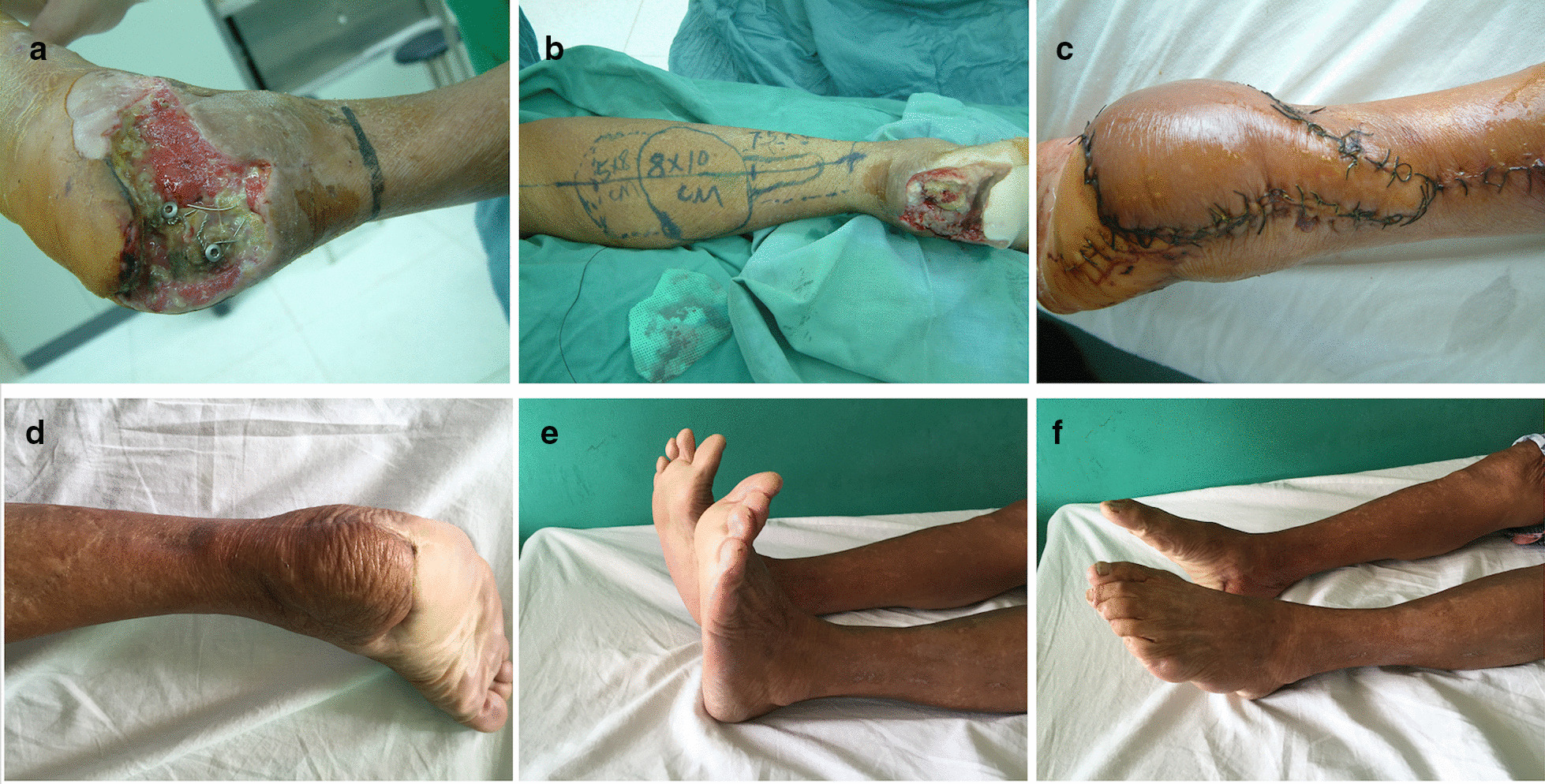
A 63-year-old female with soft-tissue defect on the heel region. Intraoperative photo showing the wound has a lot of infected necrotic tissue with internal fixations and bone exposure (**a**). A distally based sural flap was designed to cover the defect after aggressive debridement (**b**). The flap survived completely after 2 weeks (**c**). At 10 years of follow-up, the aesthetic appearance (**d**) and ankle function were excellent (**e**, **f**). The patient was able to walk independently, without claudication or obstruction in daily life

There were 53 flaps in group A, including 35 males and 18 females with a mean age of 66.7 ± 6.0 years old (ranging from 60 to 81 years old). Comorbidities included hypertension in 14 patients and diabetes mellitus in 6 patients. Seven patients have tobacco and alcohol abused. The flap size ranged from 5.0 cm × 6.0 cm to 19.5 cm × 9.5 cm. Forty-eight flaps survived uneventfully (Fig. 1), 5 flaps (9.43%) developed partial necrosis. The residual wounds are covered by skin grafting in three cases. The other two patients received under knee amputation because of the squamous carcinoma and uncontrolled infection, respectively. There was no statistically significant difference in the partial necrosis rate of flaps among the patients aged younger than 65 years (2/26, 7.69%), 65 to 70 years (1/10, 10%), and older than 70 years (2/17, 11.76%) (P = 1). The rate of partial necrosis in patients with medical comorbidities (such as smoking, diabetes mellitus and hypertension) and long-term adverse lifestyle (such as cigarette and alcohol abused) (3/21, 14.28%) was higher than that in patients without these comorbidities and lifestyle (2/32, 6.25%), though the difference is not significant (P = 0.374).

**Fig. 2:**
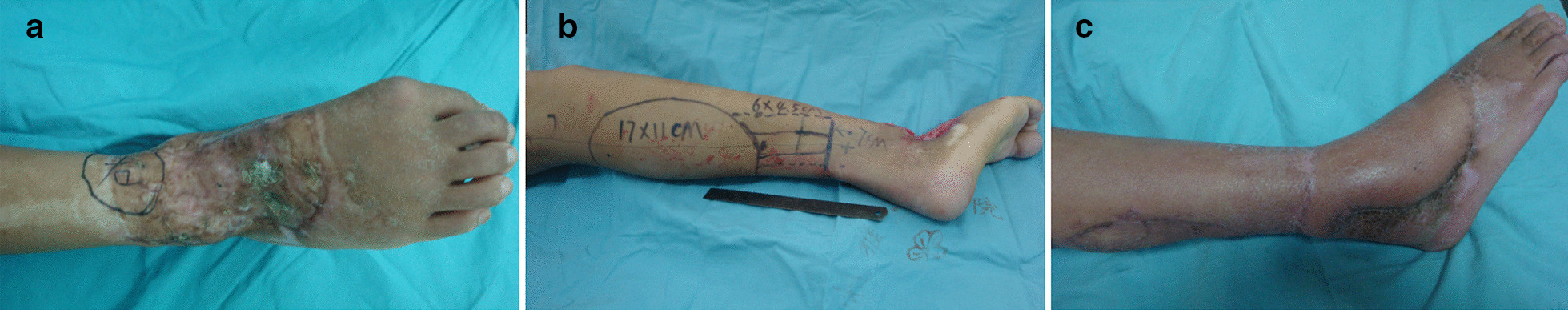
Scar covered bone on the dorsum of foot in a 30-year-old man caused by a trauma-related event (**a**). A distally based sural flap was designed to reconstruct the defect after debridement (**b**). The flap survived completely after 2 weeks. The aesthetic appearance was excellent in 14 months of follow–up (**c**)

In group B, 55 healthy young patients, including 47 males and 8 females, underwent 55 flaps, and the mean age of the patients was 24.7 ± 4.2 years old (ranging from 18 to 30 years old). All patients had no any medical comorbidities and long-term adverse lifestyle, except one who had type I diabetes and was excluded from this study. The flap size ranged from 8.0 cm × 4.0 cm to 22.0 cm × 10.5 cm. Fifty flaps survived uneventfully (Fig. 2), and partial necrosis occurred in 5 flaps (9.09%). The residual wounds are re-epithelialized by skin grafting in 4 cases, and a regional pedicled flap transposition in one case.

**Fig. 3:**
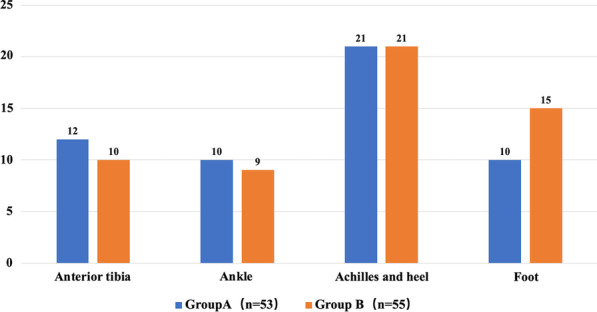
The topography of the defect site of the soft-tissue defect in group A and B

**Fig. 4:**
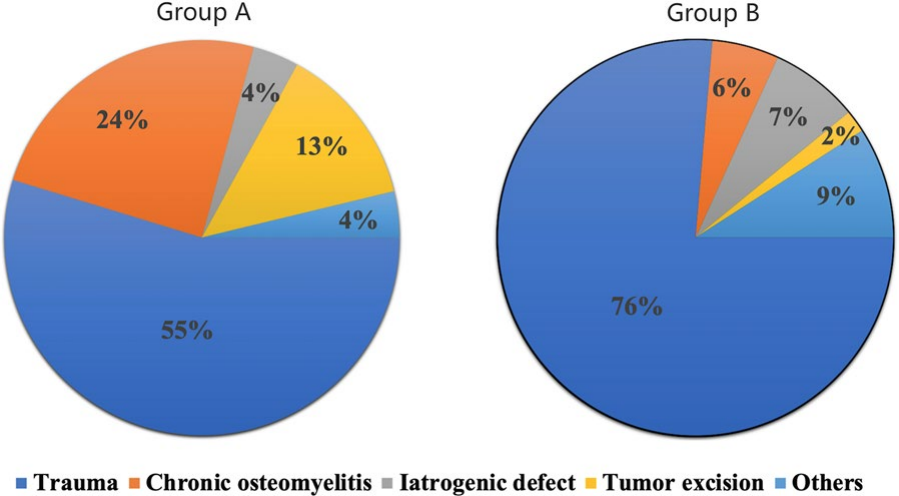
The topography of the etiology of the soft-tissue defect in group A and B

The topography of the defect site and etiology of the soft-tissue defect in group A and B are shown in Figs. 3 and 4. The main cause of the soft-tissue defects in both groups were trauma (Fig. 4), and the most defects were over the Achilles tendon and heel (Fig. 3). No significant differences were found between both groups in terms of the soft-tissue defect location (P = 0.754). The group A had more defects were caused by chronic diseases than group B, while, there were more defects due to acute etiology in group B than A, the difference in the pathogenesis of the defect were significant between group A and group B (P = 0.003). The partial necrosis rate in group A (9.43%) is higher than group B (9.09%), but the difference is not significant (P > 0.05) (Table 1). The constitute ratio of the defects that were successfully covered using the sural flap alone or combining with simple salvage method (i.e., skin grafting) is 96.22% and 98.18% in group A and B, respectively (P > 0.05) (Table 1).

**Table 1:** Comparison of the reconstruction results between groups A and B

Parameters	Group A (n = 53)	Group B (n = 55)	P
Survival condition of the flaps			1
Complete survival	48	50	
Partial necrosis	5	5	
Defect reconstruction outcome			0.614
Success^a^	51	54	
Failure^b^	2	1	

^a^Success means repair of the defect with the use of a sural flap alone or in combination with some salvage methods (i.e., skin grafting)

^b^Failure refers to amputation or reconstruction with other flaps

The risk flap factors that affected the survival of distal based sural flap [18], such as the length and width of the fasciocutaneous pedicle, length of the skin island, and total length of the flap in group A were higher than those in group B, and the pivot point site, width of the skin island, and LWR of the flap are less than those in group B, but the differences were not significant (P > 0.05) (Table 2).

**Table 2:** Comparison of flap risk factors between groups A and B

Parameters	Group A (n = 53)	Group B (n = 55)	t	P
Fascial pedicle (cm)				
Length	8.36 ± 2.38	8.32 ± 2.72	− 0.081	0.935
Width	4.19 ± 0.44	4.15 ± 0.47	− 0.488	0.627
Skin island (cm)				
Length	11.52 ± 3.57	11.42 ± 3.84	− 0.14	0.889
Width	8.27 ± 1.98	8.56 ± 2.33	0.691	0.491
Total length	19.86 ± 3.54	19.74 ± 4.17	− 0.163	0.871
LWR	4.75 ± 0.78	4.83 ± 0.98	0.452	0.652
Pivot point	7.93 ± 1.75	8.06 ± 2.31	0.326	0.754

None of the patients died during the perioperative period, nor did they suffer from life-threatening or fatal medical complications (pneumonia and pulmonary embolism, deep vein thrombosis, stroke, or heart attack). All patients in this series are received follow-up, and the period of follow-up range from 3 to 121 (mean 13.59 ± 6.37 months). The wounds of all the patients healed successfully, and the infects were controlled without recurrence in the following period. The flaps had no rupture, numbness, dehiscence, etc., and all of the patients (except two patients who amputated below the knee joint) resumed daily activities such as independent stable walking. All wounds in the donor sites healed without any complications, such as ulcer, skin graft necrosis, scar covered bone and joint contracture. All of the patients were satisfactory with the functional and aesthetic consequences.

## Discussion

With the proportion of the elderly population is increasing, the number of the elderly patients with soft-tissue defects in the distal lower extremity caused by trauma and tumor resection is increasing gradually. Reconstructions of these defect remain full of challenges.

Free tissue transplantation has opened a new era for wound repair, and the emergence of perforator flap [2], ultra-thin flap [19], chimeric flap [20] and lobulated flap [21] in the later stage has further promoted the development of free tissue transplantation. The average success rate of the free flaps reported in the literature is higher than 90%, and the free flaps have been considered as the gold standard for repairing the soft tissue defects in many institutes. The free flaps can be used to repair various types of soft tissue defects in various age groups, in line with the principle of "accurate and precise reconstruction” [22]. With the development of the microsurgery technique and the special instruments, the old age is not a contraindication to free flaps. Free flap can be successfully used to repair the soft-tissue defect in the elderly patients, and the reconstruction outcome is comparable with that in the young patients [23, 24]. However, free flap surgery has several disadvantages, such as requiring expert surgeons in the vascular separation and anastomosis, longer operation and hospitalization duration, and sacrificing a main vascular trunk in most cases [6–9]. With the degeneration of body function in elderly patients, the prolonged anesthesia and operation time will lead to many fatal medical complications, such as pulmonary infection, deep vein thrombosis, pulmonary embolism, and heart attack. Combined with the effects of nicotine use, diabetes, hypertension, atherosclerosis and other comorbidities, the risk of flap-related complications (such as vascular crisis, anastomotic embolization requiring exploration, wound dehiscence, hematoma and infection, etc.) in the elderly patients also increase [6–9].

Since its anatomy and surgical technique were described in detail by Masquelet in 1992 [10], the distally based sural flap has gradually become one of the mainstream flaps for reconstructing the soft-tissue defects of the distal lower extremity through 30 years development. Many scholars have conducted in-depth studies on the flap and come up with multifarious derived styles and technical improvements, which increasingly improved the flap viability and expanded the indications of the flap [11–18]. However, most of the studies in publications focused on the distally based sural flap being used in the young and healthy patients. Some studies have shown that the advanced age is a risk factor for partial necrosis of the flap, on account of the medical comorbidities (such as peripheral vascular disease, diabetes mellitus, hypertension, etc.) and long-term adverse lifestyle (such as nicotine inhale and alcohol abuse) [14–16]. In order to explore the reliability of the distally based sural flap employed in the elderly patients, we retrospectively reviewed the clinical data of 53 elderly patients and 55 young patients who received this flap during the same period, and the comparison between both age groups was performed.

In this study, the results show that there is no significant difference in the partial necrosis rate between the elderly group (9.43%) and the young group (9.09%). No statistically significant difference was yet found between the two group in the rate of successful wound recover using the sural flap alone or in combination with some simple remedy measures. The difference of flap factors that affected the survival of sural flap between both groups are not significant (P > 0.05). These results display that the distal based sural flap can be safely and reliably harvested to reconstruct the defect of the lower extremity in the elderly patients as well as that in the young patients. The distally based sural flap have dual blood supply and venous drainage by the peroneal vessel perforator and fascial pedicle, and the multiple true anastomosis among the adjacent peroneal vessel perforator and the nutrient vessels of both the sural nerve and the small saphenous vein may contribute to the survival of the flap in the elderly patients.

In order to improve the successful rate of the distally based sural flap in the elderly patients, many studies suggested using the delay technique to harvest the flap, with a delay time ranging from 2 to 21 days [25–27]. The delay process could promote the expansion of the choke vessels in the flap, thus increase the blood supply and venous return of the flap to improve the flap survival. But the delay surgery increases the number of operations, duration of hospitalization, economic burden and suffering of the patients. Some scholars proposed that simultaneous surgical debridement and delay surgery could reduce the times of operations and hospital stay [26], but this technique has the potential risk of donor site infection. In this series, the defect debridement and VAC coverage were implemented during the first operation, and the flap was elevated and transferred in the other scenario to cover the defect in the elderly patients. The partial necrosis rate was only 9.43%. The limb salvage was successful in 96.22% of the patients using the flap alone or combining some simple measures. Therefore, it is believed that harvesting and transferring of the distally based sural flap in the same stage is safe and reliable in the geriatric patients, and the delay procedure is not necessary.

For the young patients without comorbidity, the distally based sural flap can be harvested as a perforator pedicled propeller flap which is elevated at the suprafascial plane or raised leaving the small saphenous vein and sural nerve in situ to obtain esthetical appearance [28]. For the elderly patients, however, these maneuvers mentioned above are not adaptable, because of the peripheral vascular disease (i.e., atherosclerosis and venous insufficiency) and the malnourished integument. In the present series, the flap was raised on both the perforator and fascial pedicle as a perforator-plus fasciocutaneous flap to obtain additional artery inflow and venous drainage [29]. Meanwhile, all the flaps were elevated at the subfascial plane containing the small saphenous vein and sural nerve, not only to reduce the operation duration but also to preserve the deep fascial vascular network which is more conducive to the flap survival.

This study is a retrospective study and unable to achieve double-blind and randomization. The subsequent multi-center study can further explore the reliability of distal based sural flap harvested in the elderly population.

## Conclusion

The distally based sural flap can be effectively used to repair the soft-tissue defect of the lower extremity in the elderly patients by improving preoperative preparation, adopting appropriate surgical techniques, carefully monitoring and nursing care, and effective function exercises. It is safe and reliable to harvest and transfer the flap in one stage, and the delay surgery is not necessary.

## Data Availability

All data generated or analyzed during this study are included in this article and is available from the corresponding author upon reasonable request.

## Abbreviations

LWR: Length–width ratio
